# The Association of P300 Components With Clinical Characteristics and Efficacy of Pharmacotherapy in Alcohol Use Disorder

**DOI:** 10.3389/fpsyt.2022.770714

**Published:** 2022-03-30

**Authors:** Jing Yuan, Changjiang Wu, Li Wu, Xinxin Fan, Tingting Zeng, Li Xu, Yujun Wei, Yan Zhang, Hongxuan Wang, Ying Peng, Chuanyuan Kang, Jianzhong Yang

**Affiliations:** ^1^Department of Psychiatry, The Second Affiliated Hospital of Kunming Medical University, Kunming, China; ^2^Department of Psychosomatic Medicine, The Third People's Hospital of Qujing, Qujing, China; ^3^Department of Substance Use Disorders, The Psychiatry Hospital of Yunnan, Kunming, China; ^4^Department of Neurology, Sun Yat-sen Memorial Hospital, Sun Yat-sen University, Guangzhou, China; ^5^Department of Psychosomatic Medicine, Tongji University School of Medicine, Shanghai East Hospital, Shanghai, China

**Keywords:** alcohol use disorder (AUD), P300 components, efficacy, biological markers, pharmacotherapy

## Abstract

**Purpose:**

The purpose of this study is to explore the association of P300 components with clinical characteristics and efficacy of pharmacotherapy in alcohol use disorder (AUD).

**Methods:**

One hundred fifty-one AUD patients and 96 healthy controls were recruited and evaluated for the symptoms of depression, anxiety, sleep, and cognitive function by the Alcohol Use Disorders Identification Test (AUDIT), the 9-item Patient Health Questionnaire (PHQ-9), the 7-item Generalized Anxiety Disorder scale (GAD-7), the Pittsburgh Sleep Quality Index (PSQI), Digit Symbol Substitution test (DSST), and event-related potential P300, which is one of the averaged scalp electroencephalography responses time-locked to specific events. Among the AUD group, 101 patients finished an 8-week pharmacotherapy and were evaluated for the above data at post-intervention.

**Results:**

1. At baseline, AUD patients had higher scores of AUDIT, PHQ-9, GAD-7, PSQI, and P300 latency at Cz, Pz, and Fz and lower DSST score and smaller P300 amplitudes at Fz, Cz, and Pz compared with controls. P300 components correlated significantly with alcohol dose and score of AUDIT, PHQ-9, GAD-7, PSQI, and DSST. 2. After 8 weeks' treatment, there were significant changes for the P300 components; alcohol dose; and score of AUDIT, PHQ-9, GAD-7, PSQI, and DSST. Variables at baseline, including P300 amplitudes at Fz, Cz, and Pz; latency of Fz and Pz; alcohol dose; and scores of PHQ-9, GAD-7, PSQI, and DSST, were significantly associated with changes of reduction rate of AUDIT scores. However, P300 amplitudes at Fz, Cz, and Pz in AUD patients after 8-week treatment were still significantly shorter than healthy controls (HCs), and P300 latencies at Fz, Cz, and Pz were significantly longer than HCs. 3. When validated area under the receiver operating characteristic curve (AUC) was over 0.80, the baseline variables including amplitudes at Cz and Pz, alcohol dose, and scores of PSQI could predict the changes of reduction rate of AUDIT score.

**Conclusion:**

P300 amplitudes and latencies at Fz, Cz, and Pz could be used as biological markers for evaluating the clinical characters and severity of AUD. P300 amplitudes at Cz and Pz, sleep condition, and cognitive function at baseline could predict the efficacy of pharmacotherapy for AUD patients.

## Introduction

About 31 million people suffer from substance use disorders (SUD) worldwide (1). Among them, alcohol use disorder (AUD) is a major problem. AUD or alcoholism is defined as a problematic pattern of alcohol use accompanied by clinically significant impairment or distress (2) and now is also a severe health issue and a high economic burden on society. From the China Mental Health Survey, the weighted lifetime prevalence of AUD was 4.4%, becoming the most prevalent substance use disorder in China (3).

AUD is frequently accompanied by co-occurring psychiatric disorders or somatic disorders, such as drug use disorders, major depressive disorder, bipolar disorder I, and anxiety disorders (4). In a cross-sectional survey of 2,979 individuals with AUD, 77% reported a moderate-to-severe psychiatric or somatic disorder. Those with both AUD and a psychiatric or somatic disorder had poorer associated health-related quality of life and lower work productivity than those with AUD only (5). Due to the effects of alcohol on brain structures and subsequent alterations in neuronal function, alcohol has sedative and detrimental effects on both performance and cognitive functioning (6), resulting eventually in neurodegeneration and cognitive impairment (7).

Electrophysiological variables may represent sensitive biomarkers of vulnerability to, or endophenotypes for, AUD (8). Event-related potentials (ERPs) are averaged scalp electroencephalography responses time-locked to specific events in a sensory, motor, or cognitive task. The averaged responses or waveforms of ERP are composed of characteristic negative and positive deflections. Amplitude (μV) is defined as the difference between the mean prestimulus baseline voltage and the largest positive-going peak of the ERP waveform within a time window. Latency (ms) is defined as the time from stimulus onset to the point of maximum positive amplitude within a time window (9). One component of ERP, P300 is a large, long-lasting component observed between 300 and 700 ms at central-parietal sites after onset of visual or auditory stimuli (10). In the detection of P300, oddball paradigms are widely used to evaluate cognition. The traditional two-stimulus oddball presents an infrequent target in a background of frequent standard stimuli (9). The P300 waveform includes P3a and P3b. P3a is elicited by novel or non-target stimuli of the traditional oddball task, whereas P3b is elicited by target stimuli of the traditional oddball task (11). The P300 has been associated with a wide range of attention, memory, and premotor decisional processes, along with response inhibition, which is a common characteristic and impaired in AUD (12, 13). In some studies, reduced amplitudes and increased latencies of the P300 component compared to control subjects have been found in AUD patients (14, 15). Variables of the P300 component have also been associated with an impaired behavior control and a higher relapse probability (9, 16). As subcomponents of P300, amplitudes of P3a and P3b were found reduced in AUD patients (11, 17). Now this evidence indicates that the P300 component is considered a potential endophenotype for vulnerability to develop AUD, a predictor and biomarker for the relapse risk after alcohol withdrawal (18).

A previous study found that the relationship of prolonged P300 latency and reduced P3 amplitude with impairment of auditory information processing in schizophrenia was not influenced by neuroleptic medication (19). In another study of antidepressant response, greater baseline P3a/b amplitudes were associated with a positive antidepressant response (20). In a recent study, compared to healthy controls, AUD patients presented reductions of P3a/3b amplitude, and, after 4-week alcohol abstinence, although P3a/3b amplitudes were improved, they were still lower than those of healthy controls (11). Given the important role of P300 amplitudes and latencies in AUD, could these components reflect the efficacy of treatment in AUD? Up to now, there has been little research into the P300 component in the treatment of AUD; therefore, in this study, we aimed to explore the association of P300 amplitudes and latencies with the efficacy of treatment in AUD. We hypothesized that AUD severity, negative emotion (depression and anxiety), cognitive function, and worse treatment response would be associated with a blunted and delayed P3. We also hypothesized that P300 amplitudes and latencies would change with treatment but would still be worse than those of healthy controls.

## Materials and Methods

### Subjects

A cohort of 151 subjects of male AUD was recruited from the Second Affiliated Hospital to Kunming Medical University, Mental Health Hospital of Qujing, and Mental Health Hospital of Yunnan Province. All patients met the criteria for Diagnosis and Statistics of Mental Disorder 5th edition (DSM-V) for AUD with normal vision and hearing or within the normal range after correction and were right handed. The exclusion criteria were (1) diagnosis of other substance use disorders; (2) history of head injury, neurological disorders, or loss of consciousness; (3) history of diabetes, stroke, or hypertension that required medical intervention; (4) clinical evidence of Wernicke–Korsakoff syndrome; (5) having evidence of mental retardation and any pervasive developmental disorder; (6) having comorbid diagnoses of schizophrenia, obsessive compulsive disorder, and post-traumatic stress disorder because comorbid AUD and PTSD or OCD could lead to a high rate of treatment dropout (21, 22); (7) having serious suicidal ideation or serious attempted suicide. AUD patients were also interviewed on their lifetime use of alcohol and abused substances. These procedures yielded alcohol average dose, alcohol peak dose, and duration; and (8) having no more than 50 trials in the target or non-target averages.

A group of 96 male healthy controls (HC) were recruited from the Health Management Center of the Second Affiliated Hospital to Kunming Medical University or local community. For HC, the exclusion criteria included (1) current substance abuse or dependence; (2) history of substance dependence; (3) a current or previous mental illness (or/and treatment); (4) history of head injury, neurological disorders, or loss of consciousness; (5) without normal vision or hearing or out of range after correction; (6) history of diabetes, stroke, or hypertension that required medical intervention; and (7) left handed.

The study was carried out in accordance with the latest version of the Declaration of Helsinki and was approved by the Ethics Committee of the Second Affiliated Hospital to Kunming Medical University. Informed consent of the participants was obtained after the nature of the procedures had been fully explained. The registration number of this study is NTC 03910686.

### Measures

#### The Alcohol Use Disorders Identification Test

The Alcohol Use Disorders Identification Test (AUDIT) (23, 24) is a 10-item alcohol-screening questionnaire that was specifically designed to avoid cultural bias. The instrument had been validated (25) and used in Chinese alcohol research (26). The cutoff of AUDIT in China was determined as 7, and the higher the score, the more severe the symptoms of AUD (25).

#### The 9-Item Patient Health Questionnaire, the 7-Item Generalized Anxiety Disorder Scale, and the Pittsburgh Sleep Quality Index

Chinese versions of the 9-item Patient Health Questionnaire (PHQ-9), 7-item Generalized Anxiety Disorder scale (GAD-7), and Pittsburgh Sleep Quality Index (PSQI) with established reliability and validity were used to assess the severity of depression, anxiety, and insomnia. Subjects completed three self-administered scales: PHQ-9 (27), GAD-7 (28), and PSQI (29).

Symptom severity was defined as mild, moderate, or severe using the following cutoffs: scores of 6, 12, and 15 on the PHQ-9 for depression (30) and 4, 9, and 12 on the GAD-7 for generalized anxiety (31). Since there are no established severity cutoffs for the Chinese version of the PSQI, the continuous score of the instrument was used to establish severity, with higher scores indicating worse sleep quality.

#### Digit Symbol Substitution Test

The Digit Symbol Substitution Test (DSST) is a valid measure of cognitive dysfunction impacted by many domains, including motor speed, attention, processing speed, executive functioning, and working memory, and is sensitive to the presence of cognitive dysfunction as well as to change in cognitive function across a wide range of clinical populations (32, 33). DSST had been used in clinical neuropsychology in China (34, 35). Because there is no gold standard regarding the threshold score for which the DSST indicates cognitive impairment, the lowest quartile in the study ( ≤ 28 points) was defined poor cognitive performance, or DSST impairment, consistent with methods previously published (36).

#### ERP P300 Recording

P300 was detected by the same methods as our previous reports (37).

### Paradigm

An auditory oddball paradigm with 80% non-target stimuli (540 tones, 1,000 Hz) and 20% target stimuli (135 tones, 2,000 Hz) presented binaurally through headphones in a pseudo randomized order was used (80 dB SPL, 40-ms duration with 10-ms rise-and-fall time, and inter stimulus interval 1.5 s). Subjects were seated with their eyes closed in a reclining chair and had to press a button with their dominant hand after target stimuli. P300 recordings were measured automatically, and the researcher of P300 was blind to disease status and genotype status.

### Recording

Recording took place in a sound-attenuated and electrically shielded room adjacent to the recording apparatus (Niclolet MEGA). Subjects were seated with closed eyes in a slightly reclined chair with a head rest. Evoked potentials were recorded with electrodes Fz, Cz, and Pz. The reference electrodes were A1 and A2. A1 and A2 were linked. The electrodes were positioned according to the International 10/20 system. Fpz served as ground. Electrode impedance was <10 kΩ. Data were collected with a sampling rate of 250 Hz and an analogous band pass filter (0.16–50 Hz). After channels were selected (Fz, Cz, and Pz), data were averaged per condition (target vs. non-target) in which only segments with correct behavioral responses were included in the average and represented graphically in terms of latency (*x*-axis) and amplitude (*y*-axis). The P300 component was identified as the most positive component within the latency window of 250–600 ms. For artifact suppression, an amplitude criterion has been used (77 mV) involving three channels at any time point during the averaging period. Only wave shapes based on at least 50 averages were accepted. The P300 amplitude and latency at all electrodes for the target stimulus were determined automatically after detection by the computerized program.

### Procedure

All 151 AUD patients and 96 controls were evaluated by using AUDIT, PHQ-9, GAD-7, PSQI, DSST, and P300 detection after they were recruited at the baseline. For the AUD patients, as they finished these assessments, they were invited to attend an 8-week in-patient pharmacotherapy intervention after they gave informed consent for the research. Then, at the end of 8 weeks, they were evaluated again for the above psychological assessments and P300. In this study, all the AUD patients did not receive any kind of concurrent psychosocial treatment.

The primary outcomes were the changes of average alcohol dose/day and AUDIT scores from baseline to end point after an 8-week pharmacotherapy intervention. The second outcomes were the changes of PHQ-9, GAD-7, PSQI, DSST, and P300 from baseline to the 8-week end point after pharmacotherapy intervention.

### Pharmacotherapy Intervention Protocol

According to the treatment recommendations in the recently issued “Guidelines for the Diagnosis and Treatment of Mental Disorders in China (2020)” (38), benzodiazepine substitution therapy is recommended for AUD patients, especially for the patients of alcohol dependence. In the guidelines, the recommended dose of benzodiazepine is 10 mg at the beginning, and the highest end of the recommended range is 30–40 mg/day. The duration of treatment is from 5 days to 2 weeks, and then, the dose is gradually decreased and finally stopped in order to avoid the dependence on benzodiazepine. In addition, antipsychotics or antidepressants could be prescribed if the patients have co-occurring psychiatric disorders or psychiatric symptoms. When psychiatric symptoms persist despite a substantial reduction or cessation in drinking, the optimal approach is to continue the alcohol pharmacotherapy and add a specific psychiatric medication (39). Therefore, in this pharmacotherapy intervention, patients could be also given antidepressants if they had persistent symptoms, such as depression or anxiety symptoms.

According to the Guidelines for the Diagnosis and Treatment of Mental Disorders in China (2020), all AUD patients were prescribed benzodiazepine when they finished the initial assessment. Titration was allowed for the first week, and all subjects were treated from doses in the lower end of the recommended range, 10 mg/day, to the highest end of the recommended range, 30 mg/day. The benzodiazepine dose was allowed to decrease in response to emergence of side effects, such as excessive sedation and difficulty breathing. The titrated dose was then kept constant for 2 weeks. From the fourth week to the end of the fifth week, in order to avoid dependence on benzodiazepine, the dose was decreased from the titrated dose to withdrawal.

Antidepressant drugs were prescribed for AUD patients with comorbid major depressive disorder (MDD) or GAD according to local best practice. Titration was allowed for the first 2 weeks, and all subjects were initially treated with doses in the lower end of the recommended ranges (fluoxetine: 10 mg/day, paroxetine: 10 mg/day, sertraline: 25 mg/day, venlafaxine-XR: 37.5 mg/day, and duloxetine hydrochloride: 15 mg/day). The dose was increased after the first week. In exceptional cases (i.e., for subjects showing no serious response such as nausea, vomiting, diarrhea, indigestion, and other side effects observed at usual clinical prescribing dose), the dose could then be increased further at the second weekend, then up to a maximum daily dose at the higher end of recommended ranges (fluoxetine: 60 mg/day, paroxetine: 60 mg/day, sertraline: 200 mg/day, venlafaxine-XR: 225 mg/day, and duloxetine hydrochloride: 120 mg/day). The dose was then kept constant for 8 weeks.

### Statistical Analysis

Statistical analyses were performed by SPSS 25.0 (Statistical Package for Social Sciences, IBM, Armonk, NY) and MedCalc Statistics v19.6.3 (MedCalc Software bvba, Ostend, Belgium). Continuous data were presented as mean ± standard deviation (SD) or median (interquartile range). Categorical data were presented as absolute numbers and percentages. The expectation maximization (EM) method was used in missing data imputation. There were 19 random missing data of PHQ-9, GAD-7, PSQI, and DSST from drop-outs (38%), which were filled by the EM method.

A multi-level model was used to compare the outcome variables between pre-treatment and post-treatment as the outcome variable at level 1 and the participant at level 2.

Associations between changes in ERP data and symptoms were analyzed by Spearman correlation analysis. Univariate and multivariate linear regressions were analyzed using the reduction rate of AUDIT as the dependent variable, while the pre-intervention ERP data and psychometric scales as the independent variables.

Based on the frequencies and distributions of the reduction rate of AUDIT scores [reduction rate = (pre-treatment score – post-treatment score) / pre-treatment score × 100%], a better therapeutic effect on alcohol dependence in this research was defined as the reduction rate of AUDIT score of ≥20%, which was around the second quartile, and accordingly, the patients were classified into the effective group (*n* = 53, 52.5%) and the ineffective group (*n* = 48, 47.5%). Binary logistic regression analysis was used to explore if changes in ERP data and psychometric scales could predict a better therapeutic effect on alcohol dependence. We used a receiver operating characteristic (ROC) curve to compare the overall accuracy of the variables associated with better therapeutic effect. The area under the ROC curve (AUC) and 95% confidence interval (CI) were calculated. The larger the AUC is, the better the overall diagnostic accuracy is. The comparisons between ROC curves were performed by the DeLong method. We used a two-sided α of 0.05 for statistical significance.

## Results

### Demographic and Clinical Characteristics of AUD and HC Groups at Baseline

There were 151 AUD patients and 96 HC recruited at the baseline. As shown in Table 1, the average age of AUD was 52 ± 8 years, which was significantly older than HC. So, in the analysis of DSST and P300, age was used as a covariant to exclude the effect of age on cognitive differences. The AUD group had a higher rate of divorce than HCs. There were 14 subjects in the AUD group who had a positive family history for mental illnesses. In the AUD group, there were 79 subjects in working status, 40 subjects who were retired, and 32 subjects in unemployed status. Among the total 151 subjects in the AUD group, 32 subjects had received treatment for alcoholism.

**Table 1:** Comparison of clinical and ERP data between control group and alcoholic group.

**Variable**	**Control group (*n* = 96)**	**Alcoholic group (*n* =1 51)**		***P* value**
Age	45.2 ± 7.1	51.9 ± 8.0	*T* = −6.676	<0.001
Education years	14 [4]	15 [4]	*Z* = −1.710	0.087
Marriage			*X^2^* = 11.179	0.001
Married	89 (92.7)	115 (76.2)		
Divorced	7 (7.3)	36 (23.8)		
Dose (ml/day)	–	150 [100]		
Drink duration (y)	–	15 [12]		
PHQ-9	3.26 ± 0.65	15.63 ± 1.87	*T* = −62.42	<0.001
GAD-7	2.89 ± 0.64	15.24 ± 1.89	*T* = −61.77	<0.001
PSQI	3.46 ± 0.59	13.63 ± 1.79	*T* = −53.81	<0.001
DSST	33.64 ± 2.15	18.27 ± 2.89	*T* = 42.83	<0.001
Amplitude (μV)
Fz	14.62 [1.17]	9.12 [0.47]	*Z* = 13.009	<0.001
Cz	13.17 [1.71]	8.76 [0.95]	*Z* = 13.168	<0.001
Pz	11.38 [1.26]	8.67 [0.95]	*Z* = 12.466	<0.001
Latency (ms)
Fz	320 [46]	432 [34]	*Z* = −12.734	<0.001
Cz	334 ± 48	435 ± 29	*T* = −18.503	<0.001
Pz	336 [61]	446 [51]	*Z* = −12.422	<0.001

*Data are presented as mean ± SD, median [interquartile range] or absolute numbers (percentage)*.

In the AUD patients, according to the symptom severity of PHQ-9 at baseline, there were 85 cases comorbid with depression symptoms to a severe degree (56.3%), 36 cases with moderate depression (23.8 %), 14 cases with mild depression (9%), and 16 cases without symptoms of depression (10.9%). According to symptom severity from GAD-7, there were 82 cases comorbid with anxiety symptoms to a severe degree (54.3%), 28 cases with moderate anxiety (18.5%), 15 cases with mild anxiety (9.9%), and 26 cases without symptoms of anxiety (17.3%). The score of PSQI in AUD was significantly higher than that of HCs. The score of DSST in the AUD group was significantly lower than that of the HC group.

Also shown in Table 1, P300 amplitudes at Fz, Cz, and Pz in the AUD group were significantly smaller than those in the HC group. P300 latency at Fz, Cz, and Pz in the AUD group was also significantly longer than that of the HC group. Both in the AUD and HC groups, the amplitude was the largest and latency was the shortest for Fz among the three electrodes.

### The Relationship of P300 Data With Other Clinical Data in AUD Patients

As shown in Tables 2, 3, Spearman correlation analysis after Bonferroni correction showed that both for all AUD patients at baseline and for the patients after pharmacotherapy intervention, P300 amplitudes at Fz, Cz, and Pz correlated negatively with average alcohol dose; drinking duration; and scores of PHQ-9, GAD-7, and PSQI, but correlated positively with the score of DSST. P300 latency at Fz, Cz, and Pz correlated positively with average alcohol dose; drinking duration; and scores of PHQ-9, GAD-7, and PSQI, but correlated negatively with the score of DSST.

**Table 2:** The association between ERP data and clinical data by Spearman simple correlation in all included AUD patients by Bonferroni correction (*N* = 151).

	**Am-Fz**	**Am-Cz**	**Am-Pz**	**La-Fz**	**La-Cz**	**La-Pz**
Dose	−0.470*	−0.694*	−0.699*	0.576*	0.438*	0.494*
Drink duration	−0.498*	−0.819*	−0.857*	0.574*	0.504*	0.519*
PHQ-9	−0.614*	−0.450*	−0.463*	0.395*	0.494*	0.395*
GAD-7	−0.548*	−0.448*	−0.501*	0.473*	0.445*	0.421*
PSQI	−0.392*	−0.447*	−0.500*	0.355*	0.311*	0.344*
DSST	0.622*	0.579*	−0.609*	−0.530*	−0.563*	−0.498*

Bonferroni correction:α = 0.05/6 = 0.008, ^*^p < 0.008.

**Table 3:** The association between ERP data and clinical data by Spearman simple correlation in follow-up AUD patients by Bonferroni correction (*N* = 101).

	**Am-Fz**	**Am-Cz**	**Am-Pz**	**La-Fz**	**La-Cz**	**La-Pz**
Dose	−0.653*	−0.748*	−0.740*	0.683*	0.595*	0.599*
Drink duration	−0.623*	−0.759*	−0.778*	0.658*	0.626*	0.566*
PHQ-9	−0.603*	−0.492*	−0.511*	0.490*	0.621*	0.466*
GAD-7	−0.589*	−0.498*	−0.534*	0.533*	0.520*	0.404*
PSQI	−0.582*	−0.571*	−0.603*	0.605*	0.548*	0.453*
DSST	0.748*	0.753*	0.793*	−0.689*	−0.703*	−0.624*
Reduction rate of AUDIT	0.593*	0.652*	0.688*	−0.584*	−0.484*	−0.549*

Bonferroni correction: α = 0.05/7 = 0.007, ^*^p < 0.007.

### Therapeutic Effects of Pharmacotherapy Intervention on Average Alcohol Dose and Scores of AUDIT, PHQ-9, GAD-7, PSQI, and P300 in the AUD Patients

In total, there were 101 patients who finished the 8-week pharmacotherapy intervention. In the pharmacotherapy intervention, the average dose of benzodiazepine each day was 20.72 ± 8.64 mg/day, and the average daily dose for each antidepressant was consistent with usual clinical prescribing practices (fluoxetine: 30 ± 9 mg/day, paroxetine: 30 ± 10 mg/day, sertraline: 75 ± 25 mg/day, venlafaxine-XR: 115 ± 30 mg/day, and duloxetine hydrochloride: 60 ± 30 mg/day).

Except for the latencies of Fz and Cz, there were no significant differences for age; education; marriage status; drinking dose; and symptom duration of PHQ-9, GAD-7, and PSQI; scores of DSST; amplitudes of Fz, Cz, and Pz; and latency of Pz between completers and dropouts of the AUD group, as shown in Table 4.

**Table 4:** Comparison of completers and dropouts of AUD group.

**Variable**	**Completers (*n* = 101)**	**Drop-outs (*n* = 50)**	**Statistical value**	***P***
Age	51.9 ± 7.9	51.7 ± 8.3	*T* = 0.144	0.886
Education years	15 [4]	15 [4]	*Z* = −0.141	0.888
Marriage			*X^2^* = 0.140	0.709
Married	76 (75.2)	39 (78)		
Divorced	25 (24.8)	11 (22)		
Dose (ml/day)	150 [100]	160 [100]	*Z* = −0.558	0.577
Drink duration (y)	15 [12]	15 [13]	*Z* = −0.389	0.697
PHQ-9	15.8 ± 1.7	15.9 [3.0]	*Z* = −0.740	0.459
GAD-7	16.0 [3.0]	15.3 [2.0]	*Z* = −0.989	0.323
PSQI	13.7 ± 1.8	14.0 [2.0]	*Z* = −0.802	0.423
DSST	18.5 ± 3.2	17.9 ± 2.3	*T* = 1.079	0.282
Amplitude (μV)
Fz	9.01 ± 0.47	9.21 ± 0.39	*T* = −1.609	0.110
Cz	8.74 [0.96]	8.88 ± 0.61	*Z* = −0.836	0.403
Pz	8.65 [0.89]	8.90 ± 0.73	*Z* = −0.789	0.430
Latency (ms)
Fz	425.3 ± 22.7	436.9 ± 29.4	*T* = −2.666	0.009*
Cz	425 (38)	443.5 ± 33.8	*Z* = −2.163	0.031*
Pz	443.9 ± 31.1	445.0 ± 34.2	*T* = −0.201	0.841

^*^p < 0.05.

**Figure 1:**
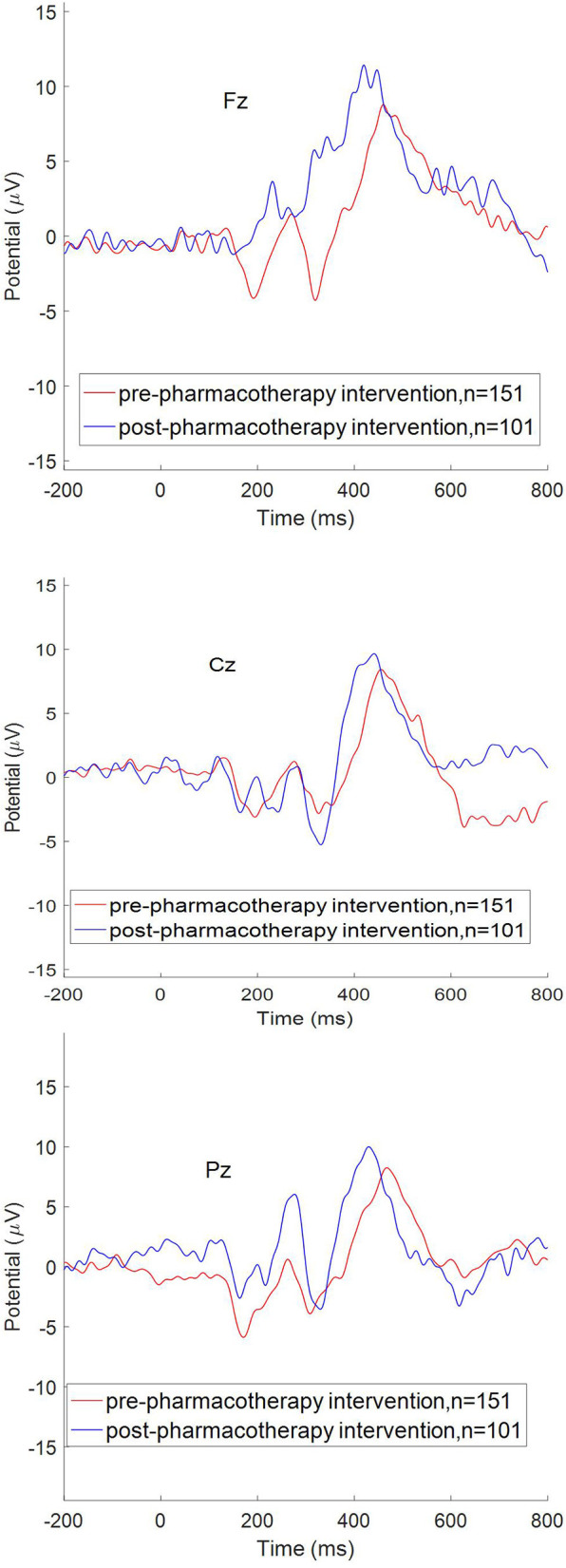
P300 (Fz Cz Pz) pre-pharmacotherapy intervention and post.

As shown in Table 5 and Figure 1, after an 8-week treatment, average alcohol dose; P300 latencies at Fz, Cz, and Pz; and scores of AUDIT, PHQ-9, GAD-7, and PSQI all decreased significantly. P300 amplitudes at Fz, Cz, and Pz and the score of DSST increased significantly. However, even after the 8-week treatment, P300 amplitudes at Fz, Cz, and Pz in AUD patients were significantly shorter than HCs, and P300 latencies at Fz, Cz, and Pz in AUD patients were significantly longer than HCs. Scores of PHQ-9, GAD-7, and PSQI were still higher than HCs, and scores of DSST after the 8-week treatment were still lower than HCs, as shown in Table 5. Table 6, by a multi-level model, also demonstrates the same results between the completer subsample (*n* = 101) as well as the entire sample (*n* = 151).

**Table 5:** Change of outcome measures after 8 weeks pharmacotherapy intervention (*n* = 101) and comparison between the post-intervention AUD group (*n* = 101) and healthy controls (HC) (*n* = 96).

**Variable**	**Pre- (*n* = 101)**	**Post- (*n* = 101)**	**HC (*n* = 96)**	**Pre- vs. post-**		**Post- vs. HC**	
				**Statistical value**	***P***	**Statistical value**	***P***
Am-Fz (μV)	9.12 [0.61]	9.89 ± 1.01	14.62 [1.17]	*Z* = −8.595	<0.001*	*Z* = 11.888	<0.001
Am-Cz (μV)	8.78 ± 0.76	9.89 [2.12]	13.17 [1.71]	*Z* = −8.725	<0.001*	*Z* = 10.788	<0.001
Am-Pz (μV)	8.74 ± 0.85	9.89 [2.19]	11.38 [1.26]	*Z* = −8.618	<0.001*	*Z* = 7.687	<0.001
La-Fz (ms)	428.1 ± 28.5	401.1 ± 33.4	320 [46]	*T* = 20.212	<0.001*	*Z* = −10.085	<0.001
La-Cz (ms)	425.0 [35.0]	399.0 [44.0]	334 ± 48	*Z* = 8.602	<0.001*	*Z* = −8.936	<0.001
La-Pz (ms)	440.0 ± 32.1	412.1 ± 39.5	336 [61]	*T* = 16.129	<0.001*	*Z* = −9.472	<0.001
Dose 1 (ml/day)	172.9 ± 59.2	90.0 [110.0]	–	*Z* = 8.563	<0.001*	–	–
Dose 2 (g/day)	99.0 [47.0]	49.5 [69.0]	–	*Z* = 8.590	<0.001*	–	–
AUDIT	34.0 [9.0]	27.0 [14.0]	–	*Z* = 8.631	<0.001*	–	–
PHQ-9	16.42 ± 2.29	9.86 ± 2.01	3.26 ± 0.65	*T* = 40.777	<0.001*	*T* = 30.680	<0.001
GAD-7	15.62 ± 1.66	9.67 ± 2.33	2.89 ± 0.64	*T* = 43.299	<0.001*	*T* = 27.537	<0.001
PSQI	14.12 ± 1.88	8.88 ± 2.50	3.46 ± 0.59	*T* = 33.956	<0.001*	*T* = 20.699	<0.001
DSST	18.41 ± 3.75	22.02 ± 4.63	33.64 ± 2.15	*T* = −22.105	<0.001*	*T* = −22.399	<0.001

Data are presented as mean ± SD or median [interquartile range]. Pre-post comparison was analyzed by paired tests, while post-HC comparison was analyzed by independent tests, and corrected by Bonferroni test: α = 0.05/2 = 0.025, *p < 0.025.

**Table 6:** The result of multi-level model analysis.

**Variable**	**Pre- (*n* = 151)**	**Post- (*n* = 101)**	***F***	***P***
Am-Fz (μV)	9.12 [0.47]	9.89 ± 1.01	51.477	<0.001*
Am-Cz (μV)	8.76 [0.95]	9.89 [2.12]	52.398	<0.001*
Am-Pz (μV)	8.67 [0.95]	9.89 [2.19]	46.328	<0.001*
La-Fz (ms)	432 (38)	401.1 ± 33.4	51.320	<0.001*
La-Cz (ms)	435 ± 29	399.0 [44.0]	67.189	<0.001*
La-Pz (ms)	446 (40)	412.1 ± 39.5	46.623	<0.001*
Dose (ml/day)	172.9 ± 59.2	90.0 [110.0]	41.779	<0.001*
PHQ-9	15.63 ± 1.87	9.86 ± 2.01	474.520	<0.001*
GAD-7	15.24 ± 1.89	9.67 ± 2.33	364.714	<0.001*
PSQI	13.63 ± 1.79	8.88 ± 2.50	247.889	<0.001*
DSST	18.27 ± 2.89	22.02 ± 4.63	45.871	<0.001*

Data are presented as mean ± SD or median [interquartile range]. ^*^p < 0.05.

As shown in Tables 7, 8, the linear regression analysis and binary logistic regression analysis exploring the therapeutic effect of pharmacotherapy confirmed the above results. P300 amplitudes at Fz, Cz, and Pz; the latency of Fz, Cz, and Pz; and scores of PHQ-9, GAD-7, PSQI, and DSST at baseline were all significantly associated with the changes of reduction rate of AUDIT scores.

**Table 7:** Factors associated with therapeutic effect on alcohol dependence by linear regression analysis (*n* = 101).

**Variables**	**Univariate analysis**				**Multivariate analysis**			
	***B***	**Beta**	***T***	***P***	***B***	**Beta**	***T***	***P***
Pre-
Am-Fz	0.158	0.542	6.422	<0.001*	–	–	–	-
Am-Cz	0.159	0.645	8.392	<0.001*	–	–	–	–
Am-Pz	0.152	0.687	9.400	<0.001*	0.122	0.552	6.541	<0.001*
La-Fz	−0.004	−0.589	−7.256	<0.001*	–	–	–	–
La-Cz	−0.003	−0.497	−5.698	<0.001*	–	–	–	–
La-Pz	−0.003	−0.537	−6.330	<0.001*	–	–	–	–
PHQ-9	−0.037	−0.457	−5.117	<0.001*	–	–	–	–
GAD-7	−0.062	−0.548	−6.519	<0.001*	−0.027	−0.244	−2.885	0.005*
PSQI	−0.050	−0.506	−5.833	<0.001*	–	–	–	–
DSST	0.032	0.652	8.554	<0.001*	–	–	–	–

*p < 0.05. Multivariate regression was analyzed using the pre-intervention variables as the independent variable and the reduction rate of AUDIT as the dependent variable by stepwise method to avoid collinearity because the independent variables were highly correlated.

**Table 8:** Factors associated with better therapeutic effect on alcohol dependence by binary logistic regression analysis (*n* = 101).

**Variables**	**Univariate analysis**		**Multivariate analysis**	
	**OR**	***P***	**OR**	***P***
Pre-
Am-Fz	3.236	<0.001*	–	–
Am-Cz	7.059	<0.001*	–	–
Am-Pz	7.356	<0.001*	4.478	0.001*
La-Fz	0.959	<0.001*	–	–
La-Cz	0.967	<0.001*	–	–
La-Pz	0.969	<0.001*	–	–
PHQ-9	0.700	0.001*	–	–
GAD-7	0.575	<0.001*	–	–
PSQI	0.405	<0.001*	0.554	0.005*
DSST	1.469	<0.001*	–	–

^*^p < 0.05. Multivariate regression was analyzed using the pre-intervention variables as the independent variable and the reduction rate of AUDIT as the dependent variable by stepwise method to avoid collinearity because the independent variables were highly correlated.

**Figure 2:**
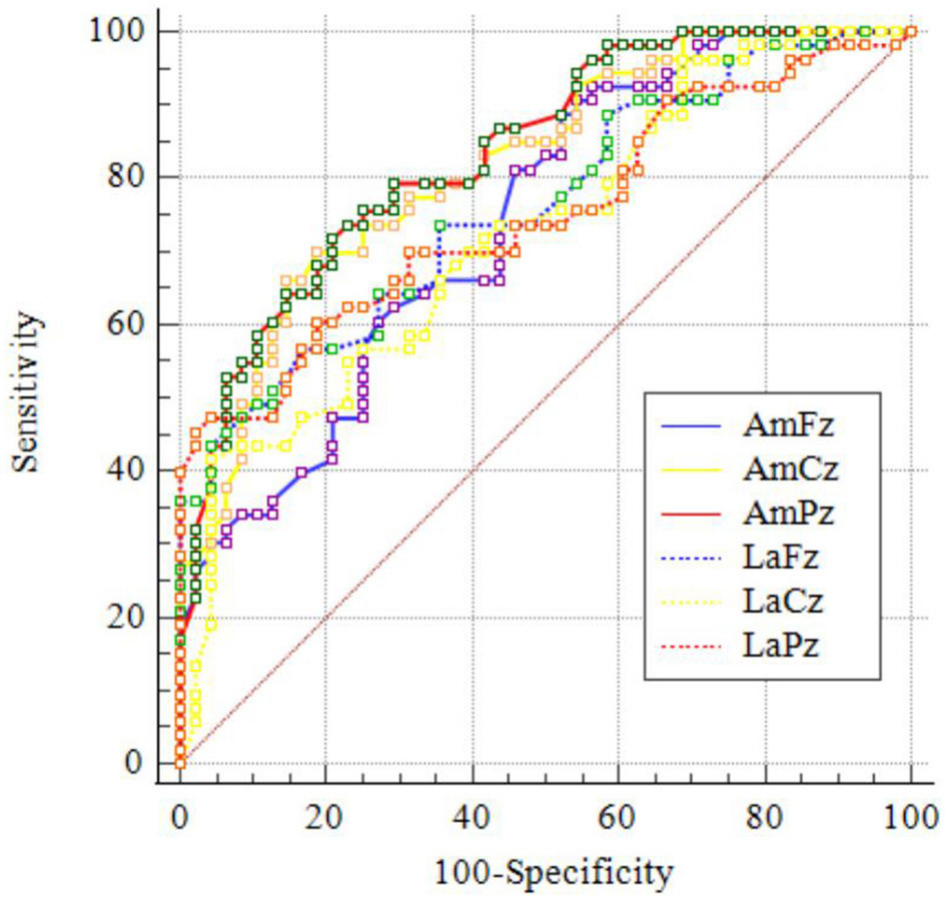
ROC curves of the ERP data in predicting the therapeutic effect.

**Figure 3:**
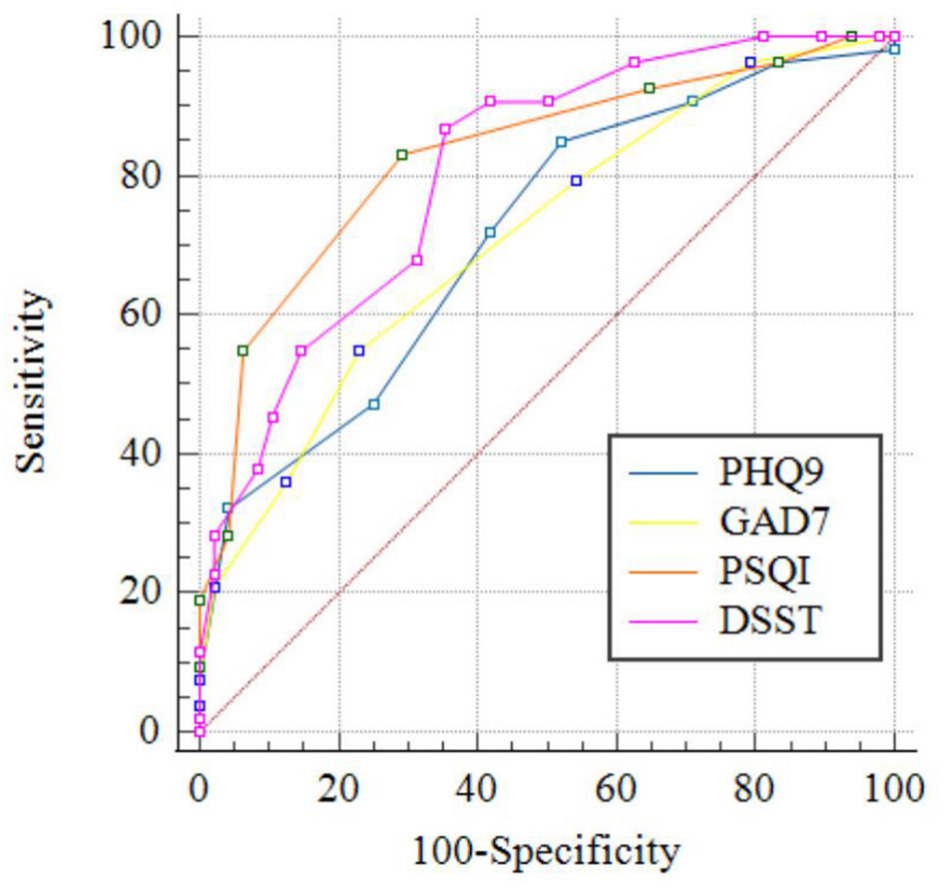
ROC curves of the clinical data in predicting the therapeutic effect.

When validated AUC was over 0.80, the variants of baseline, namely, amplitudes at Cz and Pz and scores of PSQI and DSST, could predict the reduction rate of AUDIT scores after pharmacotherapy intervention, as shown in Table 9 and Figures 2, 3.

**Table 9:** Comparison of the ROC curves for different markers in predicting the therapeutic effect.

**Variable**	**AUC**	**95%CI**	**Cut-off point**	**Se (%)**	**Sp (%)**
Am-Fz (μV)	0.741	0.645–0.823	>8.88	88.68	47.92
Am-Cz (μV)	0.817	0.728–0.887	>8.80	66.04	85.42
Am-Pz (μV)	0.835	0.747–0.901	>8.61	71.70	79.17
La-Fz (ms)	0.761	0.665–0.840	≤ 420	56.60	83.30
La-Cz (ms)	0.724	0.626–0.808	≤ 410	41.50	95.80
La-Pz (ms)	0.750	0.654–0.831	≤ 413	45.28	97.92
PHQ-9 (point)	0.715	0.616–0.800	≤ 17	84.91	47.92
GAD-7 (point)	0.713	0.614–0.798	≤ 15	54.72	77.08
PSQI (point)	0.829	0.741–0.896	≤ 14	83.02	70.83
DSST (point)	0.807	0.717–0.879	>17	86.79	64.58

*Se: The sensitivity at the cut-off point*.

*Sp: The specificity at the cut-off point*.

## Discussion

In the present study, all AUD patients had depressive or anxiety symptoms and sleep disturbances, such as insomnia. Among them, 89.1% patients were comorbid with depression, and 80.1% of them could be rated as having a moderate or severe degree of depressive symptoms. Furthermore, 82.7% patients were comorbid with anxiety, and 72.8% of them could be rated as having a moderate or severe degree of anxiety symptoms. There is considerable comorbidity between AUD and MDD or GAD (41). Results from the 2001 to 2002 US National Epidemiologic Study on Alcohol and Related Conditions (NESARC-I) showed that those with alcohol dependence had almost four times chances of having MDD than those without dependence (42). The 2012–2013 NESARC-III further indicated that those with lifetime AUD were 30% more likely to have MDD than those without AUD (4). Both moderate and severe AUD were associated with a 30% increased chance of co-occurring with MDD (41). Similarly, the comorbidity between AUD and GAD continues to generate interest. In an outpatient substance abuse clinic, nearly half of AUD patients also met criteria for current GAD, and the onset of GAD occurred prior to AUD in 67% of comorbid cases (43). In this study, the results confirmed the high comorbidity between AUD and depression or anxiety symptoms, an observation that deserves more attention. However, the results were based on a hospital survey and do not represent an epidemiological population study.

After excluding the effect of age on cognitive function, the score of DSST in the AUD group was significantly lower than that of HCs, suggesting that AUD patients had cognitive impairment. The cognitive impairment indicated by the DSST test in AUD patients was confirmed also by P300 amplitudes and latency. Both P300 amplitudes and latencies of AUD patients at Fz, Cz, and Pz were attenuated below healthy controls. The clinical factors such as depression, anxiety, sleep disturbance, and cognitive impairments found in AUD in this study were consistent with other research (17, 44).

Only a few pharmacological treatments are formally available for treating AUDs. Disulfiram, naltrexone, and acamprosate are Food and Drug Administration (FDA) approved, and nalmefene is approved in European Union (45, 46). Off-label medications, such as topiramate, baclofen, gabapentin, and ondansetron, are commonly prescribed for the treatment of AUD (47, 48). As AUD is often comorbid with different neuropsychiatric disorders, antidepressants and antipsychotics are often potential alternatives. For example, recently given its effectiveness on mood, cognition, and functioning; its good safety and tolerability profile; and its low potential for abuse, vortioxetine could represent a valid pharmacological intervention in AUD-comorbid MDD patients as part of an integrated therapeutic rehabilitation program (49).

In the “Guidelines for the Diagnosis and Treatment of Mental Disorders in China (2020)” (38), benzodiazepine substitution therapy is recommended for AUD patients, especially for patients with alcohol dependence. In this study, because most AUD patients had comorbid MDD or GAD, the combination of diazepam and antidepressants was used as the main pharmacotherapy. After the 8-week pharmacotherapy, the average alcohol dose of consumption and scores of AUDIT decreased significantly, meaning that the alcohol dependence status and severity could be improved by the combination treatment. With the improvement of AUD, other symptoms, such as depression, anxiety, and sleep disturbances, also improved concurrently.

After the 8-week pharmacotherapy, P300 amplitudes at Fz, Cz, and Pz and the score of DSST increased significantly, and P300 latencies at Fz, Cz, and Pz were significantly shorter. High reliabilities, including test–retest reliability (50), were found for the P300 amplitude and its latency in healthy monozygotic twin pairs (51) or in normal persons (52) or in treatment-naïve alcohol-dependent patients (53). Even after very prolonged abstinence, reduced P300 amplitudes were present in chronic alcoholics (54). Therefore, P300 amplitude/latency changes observed over the 8-week treatment period in this study are likely to reflect the effects of pharmacotherapy. However, scores of DSST; P300 amplitudes; and latencies at Fz, Cz, and Pz were still worse than those of HC, suggesting that the cognitive dysfunction of AUD patients had a partial recovery after the pharmacotherapy intervention, but the damage by alcohol to cognitive function would persist longer. Previous studies demonstrated that the P300 amplitude in AUD did not completely recover after prolonged abstinence and remained lower when compared to controls (55). The pharmacotherapy intervention was only 8 weeks in this study, so it might be that more treatment time is needed for recovery in cognitive function, if it is not at least partially irreversible. Recently, a review showed that antidepressants, including selective serotonin reuptake inhibitors (SSRIs), tricyclic antidepressants, mirtazapine, and venlafaxine, are promising options for their effectiveness in reducing the craving for alcohol, weekly and monthly alcohol consumption, and in inducing marked improvements in depressive and anxiety symptoms (40). Our results support this view.

Before the pharmacotherapy intervention, both P300 amplitudes and latencies of AUD patients at Fz, Cz, and Pz correlated with average alcohol dose; drinking duration; and scores of AUDIT, PHQ-9, GAD-7, PSQI, and DSST, suggesting that the P300 amplitudes and latencies at Fz, Cz, and Pz could be used as biological markers for evaluating the clinical character and severity of AUD. After the 8-week treatment, the changes of the severity of AUD demonstrated by AUDIT scores could be predicted significantly by variables at baseline, including P300 amplitudes at Cz and Pz and scores of GAD-7, PSQI, and DSST. Jaworska et al. (20) found that normal/control-like P300 amplitudes were associated with a positive antidepressant response. Insomnia is highly prevalent in patients with AUD, and it has been related to a worse course of addiction. Patients with AUD had higher prevalence of sleep-onset insomnia (56). Patients with a moderate alcohol withdrawal syndrome also presented a lower percentage of slow-wave sleep, indicating that alcohol withdrawal syndrome severity should be considered as a critical factor for the development of non-rapid eye movement sleep alterations (57). Therefore, our results corroborate these findings from the view of pharmacotherapy intervention, indicating that P300 amplitudes at Cz and Pz, anxiety, sleep condition, and cognitive function at baseline could be used as biological markers for predicting the efficacy of treatment for AUD patients.

Interpretations from this study are limited by the sample size. Although there were 151 AUD patients recruited at baseline, only 101 patients finished the 8-week intervention. Therefore, comparisons for therapeutic effects of pharmacotherapy intervention were made between the total subjects including drop-outs and completers by completer subsample analysis and ITT analysis, which showed no difference. Secondly, because of limited sample, we did not compare the effects of different antidepressants. Thirdly, in this study, most patients were comorbid with depression, anxiety symptoms, or insomnia, so these results could not be applied to AUD patients without such comorbidities. In addition, clinical characteristics such as depression, anxiety, sleep condition, and cognitive dysfunction were assessed by using self-rating scales by patients themselves; thus, there might be some subjective bias or recall error. The last limitation was the detection of P300. The P300 amplitude and latency at all electrodes for the target stimulus were determined automatically by the computerized program. However, it cannot demonstrate the data of non-target stimulus. So, the different score between P3 target and non-target could not be analyzed in this study.

In summary, the current study found that the P300 amplitudes and latencies at Fz, Cz, and Pz could be used as biological markers for evaluating the clinical characteristics and severity of AUD. P300 amplitudes at Cz and Pz, sleep condition, and cognitive function at baseline could be used as biological markers for predicting the efficacy of pharmacotherapy for AUD patients.

## Data Availability Statement

The raw data supporting the conclusions of this article will be made available by the authors, without undue reservation.

## Ethics Statement

The studies involving human participants were reviewed and approved by NTC 03910686. The patients/participants provided their written informed consent to participate in this study. Written informed consent was obtained from the individual(s) for the publication of any potentially identifiable images or data included in this article.

## Author Contributions

CK, YP, and JYa performed data collection, data analysis, data interpretation, and manuscript preparation. JYu, CW, LW, TZ, LX, YW, and HW collected the data. XF conducted statistical analyses. All authors contributed to the article and approved the submitted version.

## Funding

This work was supported by the National Key R&D Program of China (Grants 2018YFC 1314400 and 2018YFC 1314405). The grant had no further role in study design, collection, analysis, interpretation of data, writing of the report, and decision to submit the paper for publication.

## Conflict of Interest

The authors declare that the research was conducted in the absence of any commercial or financial relationships that could be construed as a potential conflict of interest.

## Publisher's Note

All claims expressed in this article are solely those of the authors and do not necessarily represent those of their affiliated organizations, or those of the publisher, the editors and the reviewers. Any product that may be evaluated in this article, or claim that may be made by its manufacturer, is not guaranteed or endorsed by the publisher.
